# The Genetics of Iron Metabolism on Biochemical and Hematological Phenotypes of Heart Failure

**DOI:** 10.3390/ijms27093778

**Published:** 2026-04-23

**Authors:** Mário Barbosa, Laura Aguiar, Ana Matias, Joana Ferreira, João Caldeira, Ana Melício, Paula Faustino, Luiz Menezes Falcão, Manuel Bicho, Ângela Inácio

**Affiliations:** 1 Instituto de Saúde Ambiental, Laboratório Associado TERRA, 1649-028 Lisboa, Portugal; 2 Departamento de Medicina Interna, Hospital Lusíadas, 1500-458 Lisboa, Portugal; 3 Medicine ULisboa for Health, Clinical Research and Innovation, Faculdade de Medicina, Universidade de Lisboa, 1649-028 Lisboa, Portugal; 4 Instituto Bento da Rocha Cabral, 1250-047 Lisboa, Portugal; 5 Departamento de Medicina II, Hospital de Santa Maria, Centro Hospitalar de Lisboa Norte, 1649-028 Lisboa, Portugal; 6 Instituto Nacional de Saúde Dr. Ricardo Jorge, 1649-016 Lisboa, Portugal; 7 Centro Cardiovascular da Universidade de Lisboa, Faculdade de Medicina, Universidade de Lisboa, 1649-028 Lisboa, Portugal

**Keywords:** heart failure, iron metabolism, association study, *HFE*, *SLC40A1*, *TMPRSS6*

## Abstract

Heart failure (HF) is frequently associated with iron deficiency and anemia, negatively impacting patient outcomes. This study aimed to investigate the contribution of genetic variation in iron metabolism-related genes to biochemical and hematological phenotypes in HF. An HF population of 182 patients with functional iron deficiency (ID) and anemia was stratified by sex and heart failure subtype, including HF with reduced ejection fraction (HFrEF) and HF with non-reduced ejection fraction (HFnrEF). Genetic variants in *HFE* (rs1799945), *SLC40A1* (rs1439816, rs2304704), and *TMPRSS6* (rs855791) were evaluated. Variants in *HFE* and *SLC40A1* were associated with differences in serum iron, ferritin, transferrin saturation, hemoglobin, and RDW. The phenotypic impact of these variants was modulated by sex and heart failure subtype, highlighting the influence of iron availability, inflammatory burden, and erythropoietic demand. In contrast, no significant associations were observed for the *TMPRSS6* variant. In conclusion, genetic variation in key regulators of iron metabolism contributes to the heterogeneity of iron-related biochemical and hematological phenotypes in HF. These findings emphasize the interplay between genetic background, sex, and heart failure physiology and support the relevance of personalized approaches to iron assessment and management in heart failure.

## 1. Introduction

Heart failure (HF) patients often have iron deficiency (ID) (absolute: serum ferritin <100 µg/L; functional: serum ferritin 100–299 µg/L with transferrin saturation <20%, according to the European Society of Cardiology (ESC) criteria) and anemia (hemoglobin <12 g/dL in women and <13 g/dL in men, according to the World Health Organization). Iron is essential for the production of hemoglobin and myoglobin, and therefore for the transport of oxygen on blood and its storage within heart muscle cells. Intracellular iron is also essential for mitochondrial energy production, such as that required for regular contraction of heart muscle [1]. The ESC guidelines recommend screening and treatment of iron deficiency in HF patients regardless of the presence of anemia [2,3].

Relative equilibrium in internal iron metabolism is usually maintained. Iron homeostasis is tightly regulated to ensure an adequate supply of iron for essential biological processes, while limiting the toxicity of excess iron [4]. The metabolism of iron in the human body involves absorption, transport, utilization, circulation, regulation, and storage [5]. After being absorbed into enterocytes, some iron is stored as ferritin, while the rest enters the bloodstream via membrane ferroportin (FPN) reaching other cell types, such as macrophages or hepatocytes, where iron can also be stored [6]. Ferroportin disease is a type of iron overload disorder caused by mutations in the *SLC40A1* gene. Hepcidin, which is produced in the liver, is the main regulator of iron balance in the body. Hepcidin and FPN work together to regulate how iron gets out of the cells [7]. When iron load increases (e.g., high iron stores, high serum iron), elevated hepcidin binds directly to membrane FPN, promoting its internalization and degradation, thereby inhibiting iron release into the blood [8]. On the other hand, hepcidin expression is reduced under hypoxic conditions, promoting iron circulation in the bloodstream. Hepcidin is upregulated by many factors, including the human haemochromatosis protein (HFE) [4]. HFE has a prevalent role as a sensor of iron levels. Hemochromatosis, a common iron overload disorder characterized by a deficit in hepcidin release or activity, is caused by mutations in *HFE* [9]. However, the most important inhibitor of hepcidin is matriptase-2, a transmembrane serine protease that is encoded by the *TMPRSS6* gene. Mutations in this gene are associated with a rare form of iron deficiency anemia that is refractory to oral iron therapy (iron refractory iron deficiency anemia, IRIDA) [10].

HF can be categorized based on the ejection fraction (EF), a measure of the heart’s efficiency in pumping blood. Heart failure with preserved ejection fraction is characterized by an EF of 50% or more, in contrast to heart failure with reduced ejection fraction (≤40%) and mildly reduced ejection fraction (40% < EF < 50%) [2]. EF can differ between sexes, being influenced by physiological and anatomical differences between male and female hearts. Research has shown that women tend to have slightly higher EF values than men, likely due to differences in heart size, structure, and hormonal factors. Women are more likely to develop heart failure with preserved ejection fraction, while men are more likely to experience heart failure with reduced ejection fraction [3,11,12].

This study aimed to evaluate the association between genetic variants in iron metabolism-related genes (*HFE*, *SLC40A1*, and *TMPRSS6*) and iron-related biochemical and hematological phenotypes in heart failure, including analyses stratified by heart failure subtype (HFrEF; EF ≤ 40 and HFnrEF; EF > 40).

## 2. Results

### 2.1. Participant Characteristics

A total of 182 participants were included, 94 female and 88 male, with a median age of 82.5 years. The characteristics of the study participants are presented in Table 1. There were 101 HFnrEF patients (64 female and 37 male, median age 84), and 81 HFrEF patients 81 (30 female and 51 male, median age 81). Cardiovascular comorbidities were frequent, particularly arterial hypertension, dyslipidemia, and hypertensive heart disease. Antiplatelet and anticoagulant therapies were commonly used.

### 2.2. HF Population Show Features of Ferropenic Anemia

Biochemical and hematological parameters of the HF population show features of ferropenic anemia (Table 2). Indeed, our HF population has funtional ID (serum ferritin 100–299 µg/L with transferrin saturation <20%) and anemia hemoglobin <12 g/dL in women and <13 g/dL in men).

### 2.3. Variation in HFE and SLC40A1 Genes Contribute to the Biochemical and Hematological Phenotypes in HF

The genetic contribution of four variants related to iron metabolism was tested regarding the biochemical and hematological phenotypes in HF in general, in HFnrEF, and in HFrEF (Table 3, Table 4, Table 5, Table 6 and Table 7). Tested variants were rs1799945 (*HFE*), rs1439816, rs2304704 (*SLC40A1*), and rs855791 (*TMPRSS6*). Genotype distributions were analyzed with the three genotypes separately, by isolating both genotypes with the variant, or by isolating the genotype homozygous for the variant. The decision to stratify the analysis by sex was based on reference values. The results are reported only when at least one analysis showed a statistically significant association; in such cases, the corresponding results for the remaining analyses are also presented, regardless of significance. Associations without significant results in any analyses were not reported.

## 3. Discussion

In this study, the HF population exhibited features consistent with functional ID and anemia. Additionally, serum iron levels were low in males and borderline in females, while RDW was elevated in males and it was borderline in females. RDW is expected to be increased in ID because newly produced red blood cells are smaller than older normal ones, leading to greater variation in cell size.

rs1799945 polymorphism in the HFE gene (C>G; H63D) was selected due to its well-established functional relevance in iron metabolism and its frequent implication in hereditary hemochromatosis, a disorder characterized by increased intestinal iron absorption and systemic iron overload [13]. Although it is considered to be a milder variant compared to C282Y, H63D has been associated with subtle but clinically meaningful alterations in iron homeostasis, making it a suitable candidate for investigating variability in iron-related phenotypes in heart failure. In our study, rs1799945 was associated with higher ferritin levels in males with HF. This finding is consistent with the known effect of this variant on iron regulation, potentially leading to increased iron availability [14]. Additionally, in women with HFrEF, rs1799945 was associated with lower RDW, a parameter often linked to a more favorable erythropoietic profile [15]. These observations may be explained by sex-related differences in lifetime iron balance. Men typically accumulate higher iron stores over time, which may amplify the phenotypic expression of HFE variants, particularly in terms of ferritin levels. In contrast, women—partly due to physiological iron loss earlier in life—tend to maintain lower cumulative iron stores, which may contribute to a more stable erythropoietic environment and lower RDW. Notably, the effect of rs1799945 on RDW appears to be context-dependent, becoming more evident in HFrEF, a condition often associated with more pronounced iron dysregulation. Increased iron availability in this setting may support more effective erythropoiesis, resulting in reduced anisocytosis.

The *SLC40A1* gene encodes ferroportin, the only known cellular iron exporter, playing a central role in systemic iron homeostasis. Variants in SLC40A1 were selected for this study due to their functional relevance in regulating iron efflux and their potential impact on circulating iron availability, an important determinant of iron status in heart failure. Pathogenic mutations in this gene are known to cause type 4 hereditary hemochromatosis (ferroportin disease), an autosomal dominant disorder of iron overload. Depending on their functional effect, these variants may lead either to iron retention within macrophages or to reduced sensitivity to hepcidin, resulting in increased iron export and systemic iron overload [16]. In line with this biological role, our findings suggest that variation in *SLC40A1* may contribute to differences in iron-related biomarkers across heart failure phenotypes. Given the key role of ferroportin in controlling iron availability for erythropoiesis and cellular metabolism, even subtle genetic variation may influence hematological parameters and iron distribution, particularly in the context of heart failure, where iron dysregulation is common. Our results are consistent with a phenotype of enhanced iron availability, showing increased hemoglobin levels in HF females, elevated serum iron in HF males, and increased transferrin saturation in HF and HFnrEF associated with the rs1439816 variant. These findings are in agreement with previous reports describing increased serum iron and ferritin levels associated with this mutation in an Italian population [17]. Regarding the sex-specific elevation of serum iron, although most male participants are likely to be in andropause, the decline in testosterone is gradual and does not abolish sex-specific differences in iron regulation. Testosterone modulates iron homeostasis primarily through the suppression of hepcidin expression and the stimulation of erythropoiesis, thereby enhancing intestinal iron absorption and mobilization of stored iron [18]. Although all women were postmenopausal, the observed increase in hemoglobin only in females may reflect lifelong differences in iron handling, with lower cumulative iron overload enabling more efficient functional iron utilization and a more effective erythropoietic response in the presence of this *SLC40A1* variant. The increase in transferrin saturation observed in HFnrEF may reflect a more permissive metabolic and inflammatory milieu, with relatively lower inflammation-associated hepcidin activity, preserved hepatic function, and more efficient iron mobilization [19]. Conversely, the greater inflammatory burden in HFrEF, associated with hepcidin induction and functional iron sequestration, may attenuate the phenotypic impact of the rs1439816 variant on transferrin saturation [20].

Concerning rs2304704, our findings suggest that the variant is consistent with the loss-of-function effect, associated with a phenotype of iron deficiency, as reflected by reduced serum iron and ferritin concentrations. While this variant does not alter the amino acid sequence of ferroportin, its location within a splice site region supports a role in modulating pre-mRNA splicing or transcript processing, potentially leading to altered ferroportin expression or function and a reduced efficiency of iron export [21]. Importantly, the phenotypic expression of this variant appears to be highly context-dependent, varying according to sex and the type of heart failure. Iron deficiency anemia is known to be more prevalent in women with heart failure with preserved ejection fraction, a population characterized by lower baseline iron stores, older age, and a high burden of comorbidities [12]. In this setting, the presence of a ferroportin loss-of-function variant may further exacerbate iron depletion, resulting in low ferritin and serum iron concentrations. In HF with preserved EF, iron is rapidly utilized by peripheral tissues and cellular metabolic pathways, and the limited capacity for cardiac output augmentation renders patients particularly sensitive to iron deficiency [22]. In HFrEF males, the same ferroportin variant is associated with higher hemoglobin levels. This observation is likely explained by a distinct pathophysiological environment in which erythropoiesis is primarily constrained by iron availability. In HFrEF, residual iron export mediated by altered ferroportin function may be sufficient to alleviate a critical bottleneck in erythropoiesis, allowing preferential delivery of iron to the bone marrow and a disproportionate increase in hemoglobin synthesis [23]. Reduced peripheral competition for iron in HFrEF may further favor this redistribution toward erythropoiesis.

Taken together, these findings support the concept that ferroportin loss-of-function variants modulate systemic iron availability, while the ultimate hematological phenotype is shaped by sex-specific iron reserves and the underlying heart failure physiology. The higher prevalence of iron deficiency anemia in women with HFnrEF provides an important clinical framework that helps contextualize and reinforce the anemia phenotype observed in this group, whereas in HFrEF the same genetic background may translate into a functionally distinct, erythropoiesis-favoring profile. This interaction between iron metabolism genetics, sex, and heart failure subtype may contribute to the heterogeneity of iron deficiency and anemia across the heart failure spectrum.

The *TMPRSS6* gene, which encodes matriptase-2, was included in this study due to its key role in the regulation of iron homeostasis through the modulation of hepcidin expression. Variants in *TMPRSS6* have been associated with alterations in systemic iron balance and are known to influence susceptibility to iron deficiency and iron-restricted erythropoiesis. Given the central role of hepcidin in the pathophysiology of iron deficiency in heart failure, this gene represents a biologically relevant candidate for investigating variability in iron-related phenotypes in this context. The lack of association observed for the *TMPRSS6* variant may reflect the complex and disease-specific regulation of iron metabolism in heart failure. Although inflammatory pathways are known to upregulate hepcidin expression, circulating hepcidin levels are frequently reduced in patients with heart failure, particularly in the presence of iron deficiency and hypoxia [24]. This distinct regulatory environment may attenuate or override *TMPRSS6*-mediated control of iron homeostasis, thereby obscuring its impact on downstream iron parameters. Moreover, *TMPRSS6* variants are generally associated with modest effect sizes and are thought to primarily influence basal hepcidin regulation rather than dynamic circulating iron indices, which may further limit their detectability in this clinical context [20,21].

**Figure 1:**
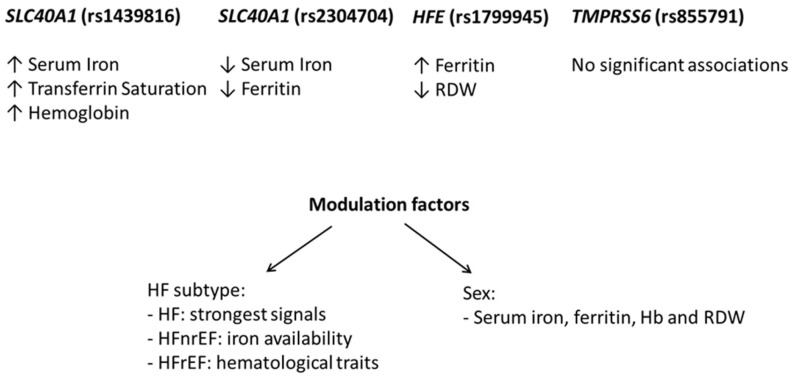
Schematic representation of the main associations between genetic variants in iron metabolism-related genes (*HFE, SLC40A1*, and *TMPRSS6*) and biochemical and hematological phenotypes in heart failure (HF). Variants in *SLC40A1* showed contrasting effects, with rs1439816 being associated with increased iron availability (higher serum iron, transferrin saturation, and hemoglobin) and rs2304704 being associated with an iron deficiency profile (lower serum iron and ferritin). The *HFE* variant (rs1799945) was associated with modulation of iron storage and erythropoiesis, reflected by increased ferritin levels and reduced RDW. No significant associations were observed for the *TMPRSS6* variant. The observed effects were modulated by sex and heart failure subtype, highlighting phenotype-specific patterns of association.

The summary of our findings is now represented in the schematic summary Figure 1.

Despite the relevance of our findings, several limitations should be acknowledged. First, the relatively modest sample size, particularly after stratification by sex and heart failure subtype, may have limited the statistical power to detect weaker genetic associations. Second, this was a cross-sectional study, precluding causal inferences regarding the relationship between genetic variants in iron metabolism and biochemical or hematological phenotypes in heart failure. Longitudinal studies would be necessary to clarify the temporal dynamics of these associations and their impact on clinical outcomes. Third, circulating hepcidin levels and inflammatory markers were not systematically available and therefore could not be integrated into the analysis, limiting mechanistic interpretation, particularly for variants affecting hepcidin–ferroportin regulation. Notwithstanding these limitations, the consistency of the observed genotype–phenotype associations across sex and heart failure subtypes supports the biological plausibility of our findings and highlights the need for larger, multicenter studies integrating genetic, inflammatory, and clinical data.

Our findings align with the ESC Heart Failure Guidelines, which recommend assessing iron and hematological status in HF patients. By shedding light on the genetic factors influencing iron metabolism in HF, this research opens avenues for developing targeted therapies and enhances strategies for risk stratification and personalized medicine.

## 4. Materials and Methods

### 4.1. Study Participants

The HF patients were followed in an Internal Medicine Department of a tertiary care university hospital. Inclusion criteria involved adult patients admitted due to decompensated HF in class III or IV of the New York Heart Association functional classification. Exclusion criteria were glomerular filtration rate less than 30 mL/min/1.73 m^2^ (calculated with MDRD formula) or undergoing renal replacement, moderate to severe liver failure (according to the Child-Pugh score), in-hospital death during the first hospitalization, hospital discharge against medical advice, and active cancer. This project included participants from the study approved by the Centro Académico de Medicina de Lisboa, report number 125/21.

### 4.2. DNA Extraction

Whole blood samples were obtained from patients and controls. Genomic DNA was isolated from total blood samples using the NZY Tissue gDNA Isolation kit, NZYTech, Lisbon, Portugal, following the manufacturer’s instructions.

### 4.3. Genotyping

The rs1799945 (*HFE*) results were obtained through ARMS. Sense primers (5′-ACATGGTTAAGGCCTGTTGC-3′) were used in combination with the specific antisense ARMS primers (5′-AGTTCGGGGCTCCACACGGCGACTCTCATG/C-3′). Amplification was initiated by hot start after 4 min at 94 °C, followed by 30 cycles of 1 min at 94 °C, 59 °C, and 72 °C. Amplicons were visualized in agarose gel where two bands of 309 bp and 171 bp were used to genotype according to Baty et al. [25].

*SLC40A1* (rs1439816, rs2304704) and *TMPRSS6* (rs855791) genotyping was obtained through EndPoint using the taqman assays, C___7476923_10, C___2108626_1_, C___3289902_10, respectively. Amplification was initiated by hot start after 10 min at 95 °C, followed by 40 cycles of 10 s at 95 °C, 1 min at 60 °C, and 1 s at 72 °C. Amplicons were visualized in the LightCycler^®^ 480 Instrument II, Roche Diagnostics GmbH, Mannheim, Germany, v1.5 software.

### 4.4. Phenotypic Characterization

Hemoglobin (Hb), mean corpuscular volume (MCV), red cell distribution width (RDW), total iron-binding capacity (TIBC), serum iron, ferritin, and transferrin saturation were collected from the patients’ hospital records.

### 4.5. Statistical Analysis

Differences between the groups were tested using Mann–Whitney test or Kruskal–Wallis non-parametric tests. All tests were performed with the SPSS 28.0 software. Statistical significance was defined as *p* < 0.05.

## 5. Conclusions

Genetic variation in *HFE* and *SLC40A1* may contribute to the heterogeneity of iron-related biochemical and hematological phenotypes observed in heart failure. Our findings suggest that the effects of these variants could be influenced by sex and heart failure subtype, potentially reflecting differences in iron availability, inflammatory burden, and erythropoietic demand. Although preliminary, these results point to a possible interaction between iron metabolism genetics and heart failure pathophysiology and may support further investigation into more personalized approaches to iron assessment and management in this context. Larger studies, including appropriate control populations, are warranted to confirm and extend these observations.

## Figures and Tables

**Table 1:** Characteristics of study participants (n = 182).

Characteristic	Value
**Age**, years, median (min.–max.)	82.5 (35–99)
**Sex**	
Female, n (%)	94 (51.6)
Male, n (%)	88 (48.4)
**Heart Failure Subtypes**	
HF with non-reduced ejection fraction, n (%)	101 (55.5)
Age, years, median (min.–max.)	84.0 (43–99)
Sex	
Female, n (%)	64 (63.4)
Male, n (%)	37 (36.6)
HF with reduced ejection fraction, n (%)	81 (44.5)
Age, years, median (min.–max.)	81 (35–94)
Sex	
Female, n (%)	30 (37.0)
Male, n (%)	51 (63.0)
**Comorbidities**	
Ischemic heart disease	
Yes, n (%)	48 (26.4)
No, n (%)	132 (72.5)
Missing, n (%)	2 (1.1)
Previous acute myocardial infarction	
Yes, n (%)	38 (20.9)
No, n (%)	142 (78.0)
Missing, n (%)	2 (1.1)
Valvular heart disease	
Yes, n (%)	60 (33.0)
No, n (%)	120 (65.9)
Missing, n (%)	2 (1.1)
Hypertensive heart disease, yes, n (%)	
Yes, n (%)	126 (69.2)
No, n (%)	54 (29.7)
Missing, n (%)	2 (1.1)
Arterial hypertension	
Yes, n (%)	172 (94.5)
No, n (%)	8 (4.4)
Missing, n (%)	2 (1.1)
Type 2 diabetes mellitus	
Yes, n (%)	80 (44.0)
No, n (%)	100 (54.9)
Missing, n (%)	2 (1.1)
Dyslipidemia	
Yes, n (%)	142 (78.0)
No, n (%)	38 (20.9)
Missing, n (%)	2 (1.1)
Smoking	
Yes, n (%)	42 (23.1)
No, n (%)	138 (75.8)
Missing, n (%)	2 (1.1)
Chronic renal failure	
Yes, n (%)	77 (42.3)
No, n (%)	103 (56.6)
Missing, n (%)	2 (1.1)
**Antiplatelet and Anticoagulant Medications**	
Acetylsalicylic acid	
Yes, n (%)	59 (32.4)
No, n (%)	120 (65.9)
Missing, n (%)	3 (1.6)
Clopidogrel	
Yes, n (%)	24 (13.2)
No, n (%)	155 (85.2)
Missing, n (%)	3 (1.6)
Ticagrelor	
Yes, n (%)	2 (1.1)
No, n (%)	177 (97.3)
Missing, n (%)	3 (1.6)
Oral anticoagulants	
Yes, n (%)	99 (54.4)
No, n (%)	80 (44.0)
Missing, n (%)	3 (1.6)

**Table 2:** Descriptive data of biochemical and hematological parameters in the HF population.

Parameters	Sex (n)	Median	Reference Values
**Serum iron (µg/dL)**	Female (92)	35.9 µg/dL	35–145 µg/dL
	Male (85)	45.4 µg/dL	50–150 µg/dL
**Ferritin (ng/mL)**	Female (92)	124.0 ng/mL	30–200 ng/mL
	Male (84)	283.5 ng/mL	30–300 ng/mL
**Transferrin saturation (%)**	Both (174)	15.0%	20–50%
**Hb (g/dL)**	Female (94)	11.3 g/dL	12–16 g/dL
	Male (88)	12.4 g/dL	14–17 g/dL
**MCV (fL)**	Both (182)	89.5 fL	80–100 fL
**RDW (%)**	Female (94)	16.0%	12.2–16.1%
	Male (88)	15.5%	11.8–14.5%
**TIBC (µg/dL)**	Both (174)	284.0	250–390

Hb—hemoglobin; MCV—mean corpuscular volume; RDW—red cell distribution width; TIBC—total iron-binding capacity.

**Table 3:** Genetic contribution to serum iron levels in patients with HF and HFnrEF.

Serum Iron							
HF		Female			Male		
Variant (Gene)	Genotype	n (%)	Median (µg/dL)	*p*	n (%)	Median (µg/dL)	*p*
**rs1439816 (*SLC40A1*)**	**GG** **GC** **CC**	68 (74) 19 (21) 5 (5)	34.5 42.8 40.5	0.885	56 (66) 26 (31) 3 (4)	37.3 45.8 63.1	**0.036**
	**CC/GC** **GG**	24 (26) 68 (74)	42.4 34.5	0.625	29 (34) 56 (66)	54.4 37.3	**0.015**
	**CC** **CG/GG**	5 (5) 87 (95)	40.5 35.4	0.796	3 (6) 44 (94)	63.1 43.9	0.151
**rs2304704** **(*SLC40A1*)**	**GG** **AG** **AA**	36 (39) 45 (49) 11 (12)	43.0 33.9 22.5	**0.048**	36 (42) 28 (33) 21 (25)	42.1 40.5 53.3	0.524
	**AA/GA** **GG**	56 (61) 36 (39)	33.5 43.0	0.178	49 (58) 36 (42)	46.4 42.1	0.769
	**AA** **GA/GG**	11 (12) 81 (88)	22.5 37.6	**0.018**	21 (25) 64 (75)	53.3 41.7	0.271
**HFnrEF**		**Female**			**Male**		
**Variant** **(Gene)**	**Genotype**	**n (%)**	**Median** **(µg/dL)**	***p***	**n (%)**	**Median** **(µg/dL)**	***p***
**rs2304704 (*SLC40A1*)**	**GG** **AG** **AA**	24 (38) 31 (49) 8 (13)	44.6 35.4 21.0	0.110	12 (32) 13 (35) 12 (32)	36.9 33.1 53.5	0.701
	**AA/GA** **GG**	39 (62) 24 (38)	33.8 44.6	0.279	25 (68) 12 (32)	45.4 36.9	0.713
	**AA** **GA/GG**	8 (13) 55 (87)	21.0 41.0	**0.041**	12 (32) 25 (68)	53.5 33.6	0.413

Significant results are shown in bold.

**Table 4:** Genetic contribution to ferritin levels in patients with HF and HFnrEF.

Ferritin							
HF		Female			Male		
Variant (Gene)	Genotype	n (%)	Median (ng/mL)	*p*	n (%)	Median (ng/mL)	*p*
**rs1799945 (*HFE*)**	**CC** **CG** **GG**	52 (60) 26 (30) 9 (10)	129.5 118.0 65.3	0.220	47 (61) 26 (34) 4 (5)	281.0 216.0 567.5	0.103
	**GG/CG** **CC**	35 (40) 52 (60)	93.8 129.5	0.237	30 (39) 47 (61)	273.0 281.0	0.921
	**GG** **CG/CC**	9 (10) 78 (90)	65.3 126.0	0.099	4 (5) 73 (95)	567.5 242.0	**0.037**
**rs2304704** **(*SLC40A1*)**	**GG** **AG** **AA**	36 (39) 45 (49) 11 (12)	121.0 164.0 58.9	**0.016**	36 (43) 27 (32) 21 (25)	309.0 204.0 361.0	0.348
	**AA/GA** **GG**	56 (61) 36 (39)	128.5 121.0	0.663	48 (57) 36 (43)	281.5 309.0	0.924
	**AA** **GA/GG**	11 (12) 81 (88)	58.9 140.0	**0.004**	21 (25) 63 (75)	361.0 228.0	0.227
**HFnrEF**		**Female**			**Male**		
**Variant** **(Gene)**	**Genotype**	**n (%)**	**Median** **(ng/mL)**	***p***	**n (%)**	**Median** **(ng/mL)**	***p***
**rs2304704** **(*SLC40A1*)**	**GG** **AG** **AA**	24 (38) 31 (49) 8 (13)	121.0 194.0 71.6	0.066	12 (32) 13 (35) 12 (32)	256.5 228.0 376.5	0.651
	**AA/GA** **GG**	39 (62) 24 (38)	140.0 121.0	0.893	25 (68) 12 (32)	361.0 256.5	0.532
	**AA** **GA/GG**	8 (13) 55 (87)	71.6 140.0	**0.029**	12 (32) 25 (68)	376.5 228.0	0.378

Significant results are shown in bold.

**Table 5:** Genetic contribution to transferrin saturation levels in patients with HF and HFnrEF.

Transferrin Saturation				
HF		Both		
Variant (Gene)	Genotype	n (%)	Median (%)	*p*
**rs1439816 (*SLC40A1*)**	**GG** **GC** **CC**	123 (71) 43 (25) 8 (5)	13.0 18.0 16.0	0.060
	**CC/GC** **GG**	51 (29) 123 (71)	17.0 13.0	**0.022**
	**CC** **CG/GG**	8 (5) 166 (95)	16.0 14.5	0.826
**HFnrEF**		**Both**		
**Variant** **(Gene)**	**Genotype**	**n (%)**	**Median** **(%)**	***p***
**rs1439816 (*SLC40A1*)**	**GG** **GC** **CC**	70 (72) 24 (25) 3 (3)	13.0 18.0 14.0	**0.042**
	**CC/GC** **GG**	27 (28) 70 (72)	18.0 13.0	**0.015**
	**CC** **CG/GG**	3 (3) 94 (97)	14.0 14.0	0.914

Significant results are shown in bold.

**Table 6:** Genetic contribution to hemoglobin levels in patients with HF and HFrEF.

Hemoglobin							
HF		Female			Male		
Variant (Gene)	Genotype	n (%)	Median (g/dL)	*p*	n (%)	Median (g/dL)	*p*
**rs1439816 (*SLC40A1*)**	**GG** **GC** **CC**	69 (73) 20 (21) 5 (5)	11.2 11.3 12.9	0.129	59 (67) 26 (30) 3 (3)	12.6 12.0 13.0	0.445
	**CC/GC** **GG**	25 (27) 69 (73)	11.7 11.2	0.248	29 (33) 59 (67)	12.3 12.6	0.246
	**CC** **CG/GG**	5 (5) 89 (95)	12.9 11.3	**0.048**	3 (3) 85 (97)	13.0 12.4	0.863
**HFrEF**		**Female**			**Male**		
**Variant** **(Gene)**	**Genotype**	**n (%)**	**Median** **(g/dL)**	***p***	**n (%)**	**Median** **(g/dL)**	***p***
**rs2304704** **(*SLC40A1*)**	**GG** **AG** **AA**	12 (40) 14 (47) 4 (13)	12.2 11.9 11.6	0.961	25 (49) 17 (33) 9 (18)	12.4 13.7 13.9	**0.005**
	**AA/GA** **GG**	18 (60) 12 (40)	11.6 12.2	0.819	26 (51) 25 (49)	13.7 12.4	**0.001**
	**AA** **GA/GG**	4 (13) 26 (87)	11.6 12.2	1.000	9 (18) 42 (82)	13.9 13.1	0.255

Significant results are shown in bold.

**Table 7:** Genetic contribution to RDW levels in patients with HFrEF.

RDW							
HFrEF		Female			Male		
Variant (Gene)	Genotype	n (%)	Median (%)	*p*	n (%)	Median (%)	*p*
**rs1799945 (*HFE*)**	**CC** **CG** **GG**	18 (67) 5 (19) 4 (15)	16.9 15.6 14.3	0.079	30 (63) 16 (33) 2 (4)	15.5 15.4 15.4	0.892
	**GG/CG** **CC**	9 (33) 18 (67)	15.2 16.9	0.106	18 (38) 30 (63)	15.4 15.5	0.647
	**GG** **CG/CC**	4 (15) 23 (85)	14.3 16.9	**0.022**	2 (4) 46 (96)	15.4 15.5	0.819

Significant results are shown in bold.

## Data Availability

The data presented in this study are available on request from the corresponding author.

## Abbreviations

The following abbreviations are used in this manuscript:

EF	ejection fraction
ESC	European Society of Cardiology
FPN	Ferroportin
Hb	Hemoglobin
HF	heart failure
HFnrEF	heart failure with no reduced ejection fraction
HFrEF	heart failure with reduced ejection fraction
ID	iron deficiency
MCV	mean corpuscular volume
RDW	red cell distribution width
SNP	single-nucleotide polymorphism
TIBC	total iron-binding capacity
WHO	World Health Organization
